# The Arabic Version of Injury-Psychological Readiness to Return to Sport Scale: Translation and Psychometric Validation

**DOI:** 10.3390/medicina61030506

**Published:** 2025-03-15

**Authors:** Osama R. Abdelraouf, Amr A. Abdel-Aziem, Nouf H. Alkhamees, Zizi M. Ibrahim, Mohamed A. Elhosiny, Shaza E. Ibrahim, Amal A. Elborady

**Affiliations:** 1 Physical Therapy Program, Batterjee Medical College, Jeddah 21442, Saudi Arabia; moh.elhosiny@outlook.com; 2 Department of Biomechanics, Faculty of Physical Therapy, Cairo University, Giza 12611, Egypt; amralmaz@tu.edu.sa (A.A.A.-A.); dr_mouly@cu.edu.eg (A.A.E.); 3 Department of Physical Therapy, College of Applied Medical Science, Taif University, Taif 21944, Saudi Arabia; 4 Department of Rehabilitation Sciences, College of Health and Rehabilitation Sciences, Princess Nourah bint Abdulrahman University, P.O. Box 84428, Riyadh 11671, Saudi Arabia; nhalkhamees@pnu.edu.sa (N.H.A.); zmibrahim@pnu.edu.sa (Z.M.I.); 5 Physical Therapy Department, Saudi German Hospital, Jeddah 21461, Saudi Arabia; pt29.jed@sghgroup.net

**Keywords:** psychological readiness, reliability, return to sport, validity, sports injuries

## Abstract

*Background and Objectives*: It is crucial to consider not only the physical variables but also the athlete’s psychological condition prior to making the decision to return to sport (RTS). It is unfortunate that there is currently no universal questionnaire available in Arabic to determine whether an athlete is ready to return to sports. So, this cross-cultural validation study was carried out to translate and validate the Injury-Psychological Readiness to Return to Sport Scale (I-PRRS) into the Arabic language. *Materials and Methods*: One hundred twenty athletes with lower extremity injuries (95 males and 25 females) completed the Arabic I-PRRS twice with a one-week time interval. An additional 50 athletes, comprising 40 males and 10 females, also participated in the study by completing the questionnaire during their initial visit to a physical therapist. Floor and ceiling effects, internal consistency, reliability, discriminate validity, convergent validity, and factor construct were analyzed. *Results*: The I-PRRS was translated into Arabic with no floor or ceiling effects. It had good internal consistency (0.84) and excellent test–retest reliability (ICC 0.88, 95% CI 0.73–0.95) where the lower bound of 95% CI indicated at least good reliability. The SEM was 1.14, and the MDC was 3.27 points. Athletes who were cleared to RTS demonstrated significant differences in their responses compared with those who were visiting their physical therapist for the first time. These differences were significant across all individual items, as well as in the total scores of the assessment (*p* < 0.001). There was a significant moderate correlation between overall scores on the I-PRRS and the Tampa Scale of Kinesiophobia (TSK) (rs = 0.69, *p* < 0.05). *Conclusions*: The Arabic version of the I-PRRS demonstrated good reliability and validity, making it a suitable tool for evaluating psychological readiness to RTS among injured athletes in Arabic-speaking populations.

## 1. Introduction

Sport and exercise are widely recognized for their benefits in enhancing physical fitness and overall health. However, the increased activity and competitiveness associated with sports also elevate the risk of injury [1]. Injured athletes often experience not only physical but also significant psychological impacts, which can hinder their recovery and return to sport (RTS) [2,3]. Research has shown that many injured athletes experience psychological distress comparable to individuals seeking treatment for mental health disorders, underscoring the profound emotional toll of sports injuries [4]. For instance, a study found that injured athletes often suffer from diminished self-efficacy and confidence, coupled with a persistent fear of re-injury, which can delay their return to play [5,6]. These psychological barriers, including negative emotions, performance anxiety, and lack of confidence, can significantly influence the rehabilitation process and overall recovery outcomes [7]. In fact, a study on athletes recovering from anterior cruciate ligament reconstruction (ACLR) revealed that those who believed their injury would negatively impact their future performance were 3.5 times less likely to return to their sport [8].

The process of returning to sport is influenced by a multitude of factors, including health-related, sport-specific, physical, and personal (both internal and external) variables [9]. While physical factors such as muscle strength and neuromuscular function are critical, they alone cannot predict an athlete’s ability to successfully RTS [10,11]. This realization has spurred increased interest in exploring additional factors, particularly psychological ones, that may influence RTS outcomes. Previous research [12,13,14] has highlighted the role of psychological health in determining an athlete’s readiness to RTS, with findings suggesting that premature return to competition can lead to fear, stress, re-injury, and even injuries to other body parts, as well as a decline in physical performance [15]. Furthermore, it has been observed that an athlete’s physical readiness to resume sports does not always align with their psychological readiness, emphasizing the need for a holistic approach to rehabilitation [16]. As a result, assessing and addressing psychological readiness has become a critical component of the rehabilitation process, particularly for athletes recovering from ACLR, where it is now considered one of the key criteria for clearance to RTS [17,18]. A recent meta-analysis and systematic review further highlighted sex differences in psychological readiness, with males being more psychologically prepared than females to resume sports following ACL reconstruction [19].

To evaluate the psychological and emotional aspects of injury, various questionnaires and scales have been developed over the years [20,21,22]. However, many of these tools provide general trait assessments, which may not fully capture the sport-specific confidence required for a successful return to sport [23]. In this context, Douglas Glazer [18] developed the Injury-Psychological Readiness to Return to Sport Scale (I-PRRS), a tool specifically designed to assess injured athletes’ psychological readiness to RTS participation, regardless of the type of injury. The I-PRRS scale, which ranges from 0 to 60, requires athletes to score at least 50 to be considered psychologically prepared for RTS. Its reliability and validity have been well documented, making it a valuable tool for clinicians and researchers [24]. The I-PRRS was chosen for this study due to its multiple advantages, including its simplicity and ease of use, sport-specific psychological assessment, applicability across various sports and injury types, and strong predictive validity [25,26].

In recent years, the I-PRRS has undergone validation in multiple languages, such as Persian; Dutch; and, more recently, Thai [17,27,28]. However, an Arabic version of the scale remains unavailable, despite the growing need for such a tool in Arabic-speaking countries. The translation and validation of the I-PRRS into Arabic could significantly enhance clinical decision-making, potentially reducing injury recurrence rates and improving rehabilitation outcomes.

Previous research indicates a notably high incidence of sports injuries among Arab athletes, with the prevalence varying based on the injury type and location. For instance, a study examining sports-related injuries in Arab countries found that muscle strains were the most common type of injury, accounting for 34% of all injuries [29]. Another study reported that 68.8% of participants experienced sports-related injuries, with football being the most common sport played [30]. An epidemiological study on sports-related injuries among athletes in Jeddah, Saudi Arabia, revealed that soccer accounted for 50% of injuries, basketball for 34%, and swimming for only 2% [31].

Another key factor is the rising participation of females in sports across Arab countries, both recreationally and competitively. The absence of an Arabic version of the I-PRRS represents a critical gap in the tools available to sports clinicians in the region. Therefore, the primary aim of this study was to assess the psychometric properties of the Arabic translation of the Injury-Psychological Readiness to Return to Sport scale. By doing so, this research sought to provide a validated tool that could aid in the psychological assessment of injured athletes in Arabic-speaking contexts, ultimately contributing to more effective rehabilitation strategies and improved RTS outcomes.

## 2. Materials and Methods

This cross-cultural validation study was carried out between March 2023 and May 2024. The I-PRRS was translated into Arabic and culturally adapted in the initial phase. Before athletes with lower extremity injuries resumed high-impact sports, the Arabic version of the I-PRRS-AR was evaluated for floor or ceiling effects, validity, and reliability. The ethical committee of Batterjee Medical College reviewed the procedures and approved the study (RES-2022-0127).

### 2.1. Scale Translation Process

The process of translating and culturally adapting the Injury-Psychological Readiness to Return to Sport (I-PRRS) scale from English to Arabic was meticulously conducted to ensure its relevance and accuracy for Arabic-speaking athletes. This adaptation adhered to the comprehensive guidelines established by Beaton et al. [32] for the cross-cultural adaptation of self-report scales.

The initial step involved the independent translation of the original English I-PRRS scale into Arabic by two bilingual translators. One translator was a native Arabic-speaking physical therapist from Saudi Arabia, while the other was an American English lecturer fluent in Arabic. This dual-translation approach aimed to capture the nuances of both languages and professional terminologies.

Following the forward translation, a panel of experts convened to evaluate the synthesized Arabic version. The panel comprised the original authors, a sport psychologist, the forward translators, and two sports physical therapists with over a decade of experience. This multidisciplinary team assessed the cultural relevance, clarity, and comprehensibility of the translated items, ensuring that the scale accurately reflected the original’s intent and was suitable for the target population.

To verify the accuracy of the translation, the synthesized Arabic version was back translated into English by two native English speakers proficient in Arabic. These translators were unaware of the study’s objectives, minimizing potential bias. The back-translated version was then compared with the original English scale to identify discrepancies and ensure semantic equivalence. If needed, a third translator was asked to reconcile the differences between the two versions.

A pretest was conducted with 15 injured athletes who had completed their rehabilitation programs and were preparing to return to sports. These participants were asked to complete the preliminary Arabic version of the I-PRRS scale and provide feedback on its clarity, its relevance, and any difficulties encountered during completion. This phase was crucial for identifying any ambiguities or cultural misinterpretations in the scale items. Based on these comments, the review team made minor adjustments, and the Arabic version of the I-PRRS-AR was finalized (Appendix A).

### 2.2. Participants

A sample of 120 athletes (95 males and 25 females) with a mean age of 26.3 ± 7.8 years participated in this study. All participants had sustained lower extremity injuries and were preparing to return to competition. The diagnosis of these musculoskeletal injuries was confirmed by the referring physician. The sample size was calculated referring to the criteria established for assessing the characteristics of health status measures, which indicated a minimum sample of 100 persons for studying reliability and 50 for validity [33]. The sample size of 50 athletes was confirmed through a statistical power analysis using G*Power 3.1.9.7. With 80% power, a 0.05 alpha level, and a medium effect size of 0.5, the analysis validated that the sample was sufficient for the study’s objectives.

The study was conducted across four sports rehabilitation clinics in Jeddah, Saudi Arabia, affiliated with BMC College. The process began by sending a request email introducing the primary investigator, outlining the study’s purpose, and detailing the scale used, while emphasizing its potential benefits to sports medicine and rehabilitation. Upon receiving approval, participant selection was carried out collaboratively between the study investigators and the clinics’ senior therapists. If an athlete met the following criteria, he/she was considered eligible: (1) they were 18 years of age or above and proficient in Arabic; (2) they were diagnosed with a lower extremity sports-related injury; (3) prior to the injury, they practiced a high-impact activity twice a week that involved jumping, turning, and changing directions; and (4) they were willing to get back to pre-injury levels of performance. Athletes were excluded if they had discontinued their sport for reasons unrelated to their reported injury, had a history of fractures or dislocations, sustained upper extremity sports injuries, or were not native Arabic speakers [27,28].

According to the routine protocol approved by the involved rehabilitation centers, the decision for an athlete’s return to sports was based on multiple tests. These included isokinetic dynamometry (to evaluate muscle strength asymmetry), single-leg hop tests (distance and triple hop), the drop jump test (for reactive strength and landing mechanics), and the star excursion balance test (SEBT). Before the study began, each athlete signed a consent form to participate. Table 1 shows demographic data of the participants.

### 2.3. Procedures

Athletes’ personal data, including demographic information such as age, sex, and sport discipline, as well as their previous training history (e.g., years of experience, training frequency, and intensity), were meticulously recorded using standardized data collection forms. To ensure the highest level of expertise in data collection, three licensed physical therapists, each with at least five years of experience in managing sports-related injuries, were recruited to oversee the study’s implementation. These therapists were responsible for administering assessments, guiding participants through the study protocols, and ensuring the consistency of data collection.

The participating athletes were asked to complete two validated self-report measures: the Injury-Psychological Readiness to Return to Sport Scale in Arabic (I-PRRS-AR) and the Tampa Scale of Kinesiophobia in Arabic (TSK-AR). These tools were selected as gold standards to explore the construct validity of the newly developed Arabic version of the scale. The I-PRRS-AR evaluates athletes’ psychological readiness to RTS, while the TSK-AR measures fear of movement or re-injury, both of which are critical factors in the rehabilitation process.

To further establish the discriminative validity of the I-PRRS-AR, it was administered to a second group of 50 athletes who had recently sustained lower extremity injuries. The sample size for this group was validated through power analysis using G*Power software (version 3.1.9.7). These athletes were recruited during their initial visit to the four specialized sports rehabilitation clinics. This step was crucial to determine whether the scale could effectively differentiate between athletes at varying stages of recovery and with differing levels of psychological readiness.

Test–retest reliability was assessed by having participants complete the I-PRRS-AR a second time, seven to ten days after the initial administration. This time interval was selected to achieve a balance between minimizing genuine changes in the construct being measured and reducing the potential for recall effects. Specifically, it was deemed sufficiently short to ensure that participants’ psychological readiness to return to sport remained relatively stable, yet long enough to prevent them from recalling their previous responses, thereby minimizing bias in the results [34].

In addition to reliability and validity testing, the scale’s internal consistency was evaluated using Cronbach’s alpha to ensure that all items within the scale measured the same underlying construct. The standard error of measurement (SEM) was also calculated to assess the precision of the scale. Furthermore, potential floor or ceiling effects were examined as these could compromise the validity and reliability of the scale. Floor or ceiling effects occurred when a significant proportion of participants scored at the lowest or highest possible ends of the scale, respectively. Such effects could limit the scale’s ability to detect meaningful differences between individuals with extremely low or high scores, as well as its sensitivity to measure changes over time [33].

### 2.4. Questionnaires

The I-PRRS scale is a concise and easy-to-administer tool designed to measure an athlete’s psychological readiness to resume their sports pre-injury level following a sports-related injury [18]. The psychometric properties of the I-PRRS scale have been reported in the literature [35]. Each of the scale’s six items has a rating between 0 and 100. Scores of 0, 50, and 100 indicate minimal, moderate, and maximum confidence, respectively, in relation to each item. The total scale score is computed by adding the scores of all six items, resulting in a raw score that is then divided by ten. This calculation yields a final score ranging from 0 to 60. This final score provides a clear indication of the athlete’s psychological readiness: a score of 60 reflects a high level of confidence, 40 indicates moderate confidence, and 20 suggests low confidence.

On the other hand, the TSK is a self-reported questionnaire developed by Knapik et al. [36] to assess fear of movement or recurrence of injury, which is a common psychological barrier among individuals recovering from injury. The TSK comprises 17 items, each rated on a four-point Likert scale ranging from 1 (“strongly disagree”) to 4 (“strongly agree”). The total score is calculated by summing the responses to all 17 items, resulting in a possible score range of 17 to 68. A score of 68 indicates a strong fear of movement or reinjury, while a score of 17 suggests minimal fear. Notably, four of the items are negatively worded and require reverse scoring to ensure accurate interpretation. Individuals with elevated scores may be more likely to avoid physical activity due to fear of pain or further injury, which can hinder rehabilitation progress and delay return to sport. The TSK has been widely validated and adapted for use in various populations and languages. For instance, Juweid et al. [37] translated the TSK into Arabic and evaluated its validity and reliability, which turn the questionnaire into a valuable instrument to assess kinesiophobia in Arabic countries. The TSK is particularly useful for identifying individuals who may benefit from targeted psychological interventions to address fear-related barriers during recovery.

Both the I-PRRS and TSK play critical roles in the holistic assessment of athletes recovering from injury. While the I-PRRS focuses on measuring psychological readiness and confidence to return to sport, the TSK provides insights into the fear of movement or reinjury that may impede recovery.

### 2.5. Statistical Analysis

The statistical analysis was carried out using IBM, SPSS for Windows, version 22 (IBM SPSS, Chicago, IL, USA). Descriptive statistics were used to express the values of the I-PRRS and athletes’ characteristics. To look at the floor and ceiling impacts, the percentages of the athletes who scored zero or the maximum were measured. If more than 15% of the athletes achieved either the lowest possible score (floor effect) or the highest possible score (ceiling effect), floor or ceiling effects were deemed to exist [33].

Internal consistency was evaluated by Cronbach’s alpha, a widely used statistical measure that assesses the reliability of a scale. A scale’s reliability is evaluated by examining the degree to which a group of items within a scale or questionnaire are interrelated. This assessment helps determine the consistency and stability of the measurement tool in capturing the intended construct. Cronbach’s alpha values range from 0 to 1, with higher inter-item correlations generally indicate greater reliability, suggesting that the items are measuring the same underlying concept effectively. Generally, an alpha coefficient between 0.70 and 0.90 is considered acceptable to good, suggesting that the items within the scale measure the same underlying construct in a reliable manner. A value below 0.70 may indicate insufficient correlation among the items, suggesting a need for scale refinement or item revision. Conversely, a value above 0.90 might indicate excessive redundancy, meaning that some items could be too similar or unnecessary [38].

Measuring the intraclass correlation coefficient (ICCa) (two-way random effects model, single measure) and its 95% confidence interval (CI) was used to assess test–retest repeatability using the first and second administrations. Excellent repeatability was indicated by an ICC of 0.75 or higher, good reliability was indicated by an ICC ranging from 0.60 to 0.74, fair reliability was indicated by an ICC between 0.40 and 0.59, and poor reliability was indicated by an ICC below 0.40 [39].

The standard error of measurement (SEM) was calculated using the formula SEM = SD × √(1 − ICC), where SD represents the standard deviation of the differences between the first and second administrations. This formula quantifies the precision of the measurement tool by estimating the variability in scores attributable to the measurement error [40]. Meanwhile, the formula MDC = SEM × 1.96 × √2 was used to calculate the minimal detectable change (MDC), where SEM represents the standard error of measurement. This calculation determines the smallest change in score that can be considered statistically significant, beyond measurement error, with 95% confidence [40]. The variances between the first and second examination were evaluated using Wilcoxon tests for all items and the overall score of the I-PRRS-AR.

To assess the factor structure of the translated scale, a confirmatory factor analysis (CFA) was conducted on all items of the I-PRRS-AR. The model’s goodness of fit was evaluated using several fit indices, including the root mean square residual (RMR), the root mean square error of approximation (RMSEA), and the comparative fit index (CFI). These indices provided a comprehensive evaluation of how well the hypothesized factor structure aligned with the observed data. The model was deemed to have an acceptable fit if the RMR was less than 0.05 and the RMSEA was below 0.08. Furthermore, a CFI value above 0.90 was considered indicative of a well-fitting model [41].

Spearman’s rank correlation coefficient was the statistical method used to compare the I-PRRS and TSK scores, and the convergent validity was assessed. A correlation of 0.90 to 1.0 was regarded as very high, 0.70–0.90 was regarded as high, 0.50–0.70 was regarded as moderate, and 0.30–0.50 was regarded as low, while 0.00–0.30 was regarded as insignificant [24]. The I-PRRS scores of athletes who returned to sport were compared with those who were visiting a physical therapist for the first time using the Mann–Whitney U test to evaluate discriminative validity. A *p*-value of less than 0.05 was considered statistically significant, indicating a meaningful difference between the two groups.

## 3. Results

### 3.1. Translation Procedure of the I-PRRS

The I-PRRS scale was translated through a rigorous forward and backward translation process, adhering to established guidelines to assure both linguistic accuracy and conceptual consistency between the English version and its Arabic adaptation.

During the back-translation review, only a few minor syntactical inconsistencies were identified in three questions. These discrepancies primarily involved differences in phrasing and subtle variations in word choice. To resolve these issues, a third independent translator was consulted to reconcile the differences between the two versions. The final wording was refined through discussions among the translators and expert panel members until a consensus was reached.

### 3.2. Characteristics of Athletes

A total of 120 Saudi athletes (95 males and 25 females) who had sustained lower extremity injuries and were preparing to return to competition were included in the current study. The mean age of the participants was 26.3 ± 7.8 years, with males comprising 79.16% of the sample. The injuries included ACL injuries requiring surgery (42 cases), knee injuries treated conservatively (20 cases), calf muscle injuries (14 cases), hamstring muscle injuries (12 cases), ankle inversion injuries (15 cases), and adductor injuries (17 cases).

The participants represented a variety of sports, including soccer (57 athletes), basketball (27 athletes), volleyball (19 athletes), handball (14 athletes), and self-defence sports (3 athletes). In terms of educational background, the distribution was as follows: high school students (18 participants), undergraduate college students (62 participants), individuals holding a college degree (27 participants), and those with a pre-college certificate (17 participants).

### 3.3. Floor or Ceiling Effects

The floor and ceiling effects were absent for the I-PRRS-AR. The I-PRRS Arabic version’s total score varied from 10 to 60 (49.75 ± 7.32) in the first administration and from 20 to 60 (51.99 ± 5.99) in the second administration. Only nine athletes (7.5%), and eleven athletes (9.1%) achieved the highest possible score of 60 in the first and second administrations, respectively. However, no athlete scored zero in both administrations of the scale.

### 3.4. Internal Consistency

The scale demonstrated good internal consistency across both administrations, with Cronbach’s alpha values of 0.84 for the first administration and 0.89 for the second administration. These results indicate a high level of reliability and consistency in the measurement of the construct being assessed. Within a median time span of 7 days, 100 out of 120 participants completed the I-PRRS-AR twice. The means for the first administration and the second administration were 49.75 ± 7.32 and 51.99 ± 5.99, respectively (*p* = 0.375).

### 3.5. Reproducibility (Test–Retest Reliability)

As shown in Table 2, all I-PRRS items showed good to excellent test–retest repeatability, with ICCa values for each item ranging from 0.69 to 0.83. The I-PRRS total score demonstrated excellent test–retest reliability, with an ICC of 0.88 (95 percent confidence interval [CI] 0.73–0.95), so at least good test–retest validity was indicated by a value of at least 0.73. The score difference between the first and second administrations was 2.24 points with an SD of 2.51 and an SEM of 1.14. The MDC was found to be 3.27 points.

### 3.6. Construct Validity

#### 3.6.1. Discriminate Validity

The results of the Mann–Whitney U test showed that athletes who initially visited their physical therapist with recent injuries had statistically significant lower scores in all questions and the overall score of the PRRS-AR (mean of 36.32 ± 8.49) than athletes who were returning to sports (mean of 49.75 ± 7.32) (*p* = 0.001), as revealed in Table 2.

#### 3.6.2. Convergent Validity

For convergent validity, Spearman’s rank correlation showed a significant (*p* < 0.05) and moderate correlation between the overall scores of the I-PRRS and the TSK (rs = 0.69). Also, fair to moderate correlations were found between the total score on the TSK and each question of the I-PRRS-AR (Table 3).

### 3.7. Factor Analysis

Using the data collected from 120 athletes and based on the one-factor model, all six items of the Arabic version of I-RRR showed a good fit to the data (RMR = 0.075, RMSEA = 0.021, CFI = 946).

## 4. Discussion

The current study aimed to translate the I-PRRS scale into Arabic and evaluate its validity and reliability. The study findings showed that the I-PRRS was successfully cross-culturally adapted into Arabic, and it exhibited good psychometric qualities. According to the scale’s scoring method, the ceiling effect for six items with scores between 95 and 100 (total scores of 57–60) and the floor effect for six items with scores between 0 and 5 (total scores of 0–3) were evaluated. The reliability and content validity of the I-PRRS-AR were shown in current study without any indication of a floor or ceiling effect. Terwee et al. reported that the translated version’s capacity for adaptation was limited when floor or ceiling effects existed, and participants with the lowest and highest scores were not differentiated from one another. Consequently, the questionnaire validity and reliability were at risk [33]. However, the floor or ceiling effects of the original English version were not reported.

The Cronbach’s alpha assessment showed good internal consistency reliability of the I-PRRS-AR, indicating that various items of the translated version delivered consistent scores. These results agreed with the original English [18] and the previously translated versions into the Dutch and Persian languages [17,27]. Good test–retest reliability indicated a test’s internal validity and ensured that an assessment tool produced consistent results [42]. ICC agreement was used rather than ICC consistency to account for the systematic error.

In the current study, the I-PRRS-AR showed excellent reproducibility, with the lower value of 95% CI showing at least good reliability in 120 athletes ready to RTS. Unfortunately, reproducibility and agreement were not reported for the original English I-PRRS. A similar degree of test–retest reliability was reported for the Persian version of the scale [27]. However, the results of Vereijken et al. [17] showed only good test–retest reliability for the Dutch I-PRRS, with a lower bound of 95% CI indicating fair reliability. This lower ICC of 0.74 could be explained by the difference in the sample size and the time in rehabilitation, which reached up to 98 weeks in the Dutch study. The I-PRRS-AR scores between tests and retests had a small mean difference, indicating minimal variance or mean bias and adequate agreement between test–retest scores.

The I-PRRS-AR version’s discriminative validity was demonstrated by the fact that initially, injured athletes’ total scores before beginning their rehabilitation program were statistically lower compared with those of the athletes who completed their rehabilitation program. Basically, this was due to the fact that worry and uncertainty about RTS occurring right away after an injury could result in low confidence, which, if left unaddressed, could rise throughout various rehabilitation program phases [43]. According to Ross et al., negative emotions and psychological uncertainty are manageable and have huge impacts on rehabilitation outcomes and readiness for RTS [44]. Yet, the discriminative validity of the first English I-PRRS has not been examined.

To determine the construct validity, significant negative correlations were demonstrated, as hypothesized, between I-PRRS-AR and TSK-AR scores. The relationships indicated that as the I-PRRS-AR scores increased, the TSK-AR scores decreased, suggesting that confidence to play and readiness psychologically to return to sport in injured athletes were happening with a positive mood and less movement fear. The original English I-PRRS was shown to be negatively correlated with the total mood disturbance (TMD) score, indicating that psychological readiness to RTS and mood states were related [18]. However, the studies of Vereijken et al. [17] and Slagers et al. [45] reported fair correlation between the total score of the Dutch I-PRRS, the Dutch ACL-RSI, and the total score of the TSK. Therefore, the authors recommended that the TSK should not be used interchangeably with any of the Dutch questionnaires. They added that the TSK measured kinesiophobia, a type of fear of movement that was distinct from the fear of reinjury and psychological readiness for RTS. The findings of these studies disagreed with those of the current study due to the differences between Dutch and Arabic linguistic characteristics.

According to the 2024 systematic review by Obradovic et al. [19], the mean psychological readiness score in eleven included studies was higher than the safe recommended score for RTS, which was only marginally higher in our study. This may be because Arab nations, which compose 5% of the world’s population, have not yet fully embraced sports psychology as an integral component of physical coaching, which was one of the primary reasons for conducting this study.

This study may be considered preliminary due to certain limitations that were beyond our control at the time of the investigation. Most of the participants (79.16%) were males, which could induce sex bias and hence limit the generalization of the study results. Cultural factors significantly influence females’ participation in sports activities in Arab countries. These factors are deeply rooted in societal norms, traditions, and religious beliefs, which shape the opportunities and barriers for females in sports. Males tend to play through pain and injury most of the time, so they may mask their symptoms to RTS sooner than females, who prioritize the long-term health effects [46]. Moreover, the study was delimited to six lower extremity common injuries, but no upper extremity injuries were included. Research indicates that functional improvement in the lower extremities tends to progress more rapidly during active rehabilitation compared with other regions. This observation highlights the responsiveness of lower limb recovery to structured therapeutic interventions [47]. The delayed motor recovery after upper extremity injuries might affect the I-PRRS-AR outcomes, which could be investigated in future studies. An additional limitation was that the groups used to assess the scale discriminant validity were unequal in size. To address this, the authors conducted a statistical power analysis, a well-established method for determining sample size adequacy. Using G*Power software version 3.1.9.7, with a statistical power of 80%, an alpha level of 0.05, and a medium effect size of 0.5, the analysis confirmed that the sample size was sufficient to meet the study’s objectives. Finally, the responsiveness of the I-PRRS scale that demonstrates the change of the score over time was not examined in this study.

## 5. Conclusions

The I-PRRS was successfully translated into the Arabic language and demonstrated excellent test–retest reliability and good internal consistency, with the lower bound of the 95% confidence interval (CI) indicating at least good reliability. The I-PRRS-AR demonstrated good discrimination and convergent validity when correlated with the TSK. The successful translation and validation of the I-PRRS into Arabic (I-PRRS-AR) allow for its practical use in assessing psychological readiness among Arabic-speaking athletes recovering from injury. Clinicians and sports professionals can confidently implement the I-PRRS-AR to guide return-to-sport decisions, monitor rehabilitation progress, and reduce reinjury rates.

## Figures and Tables

**Table 1:** Characteristics of athletes returning to sport.

		First Group (*n* = 120)	Second Group (*n* = 50)
Sex	Female, %	79.16	80%
	Male, %	20.84	20%
Age, mean ± SD (year)		26.3 ± 7.8	24.5 ± 4.2
Training sessions per week, mean ± SD		4.5 ± 2.5	3.9 ± 8.5
Number of matches per week, mean ± SD		1.7 ± 0.8	2.2 ± 0.4
Time since injury occurrence, mean ± SD (weeks)		9.6 ± 4.6	-
Time in rehabilitation, weeks		9.2 ± 3.2	-

SD, standard deviation.

**Table 2:** I-PRRS-AR scores for athletes RTS and athletes visiting physical therapists.

		ICC (95% CI)	Athletes RTS (*n* = 120), Mean ± SD	Athletes Visiting Physical Therapists for the First Time (*n* = 50), Mean ± SD
Scale 1st question	1st administration	0.76 (0.65–0.84)	85 ± 10 *	70 ± 10
	2nd administration		90 ± 10	-
Scale 2nd question	1st administration	0.74 (0.63–0.82)	75 ± 10 *	60 ± 20
	2nd administration		80 ± 10	-
Scale 3rd question	1st administration	0.81 (0.67–0.91)	85 ± 10 *	55 ± 15
	2nd administration		85 ± 15	-
Scale 4th question	1st administration	0.69 (0.53–0.75)	90 ± 10 *	70 ± 10
	2nd administration		95 ± 5	-
Scale 5th question	1st administration	0.71 (0.62–0.79)	85 ± 10 *	70 ± 20
	2nd administration		90 ± 10	-
Scale 6th question	1st administration	0.83 (0.72–0.91)	90 ± 10 *	70 ± 15
	2nd administration		90 ± 10	-
Scale total score	1st administration	0.88 (0.73–0.95)	49.75 ± 7.32	36.32 ± 8.49
	2nd administration		51.99 ± 5.99	-

I-PRRS-AR, Injury-Psychological Readiness to Return to Sport Arabic; ICC, intraclass correlation coefficient; CI, confidence interval; * significant at *p* < 0.05; SD, standard deviation.

**Table 3:** Spearman correlations between the I-PRRS-AR and the TSK-AR (*n* = 120).

	I-PRRS, Question 1	I-PRRS, Question 2	I-PRRS, Question 3	I-PRRS, Question 4	I-PRRS, Question 5	I-PRRS, Question 6	I-PRRS, Total
TSK	rs = 0.49 *	rs = 0.52 *	rs = 0.58 *	rs = 0.43 *	rs = 0.56 *	rs = 0.46 *	rs = 0.69 *

* *p* < 0.05; I-PRRS-AR, Arabic Injury-Psychological Return to Sport; TSK, Tampa Scale of Kinesiophobia.

## Data Availability

Data is available on request.

## Appendix A

*The Arabic version of Injury-Psychological Readiness to Return to Sport Scale (I-PRRS)*	
مقياس الاستعداد النفسي للعودة لممارسة الرياضة	
Please rate your confidence to return to your sport on a scale from 0–100	
no confidence at all = 0	
moderate confidence = 50	
complete confidence = 100	
الرجاء تقييم مقدار الثقة النفسية للعودة لممارسة رياضتك باستخدام مقياس من (صفر إلى 100) حيث	
غير واثق تماما للعودة لممارسة الرياضة = صفر	
ثقة متوسطة للعودة لممارسة الرياضة = 50	
واثق تماما في العودة لممارسة الرياضة = 100	
1- My overall confidence to play is …………	
2- My confidence to play without pain is………….	
3- My confidence to give 100% effort is………………….	
4- My confidence to not concentrate on the injury is…………	
5- My confidence in the injured body part to handle to demands of the situation is……….	
6- My confidence in my skill level/ability is …………	
……….…………….. مقياس ثقتي بصورة عامة في العودة لممارسة اللعب هو	-1
……….……………………………… مقياس ثقتي في اللعب بدون ألم هو	-2
……………….. مقياس ثقتي في بذل 100 % من مجهودي البدني أثناء اللعب هو	-3
………………………………. مقياس ثقتي في عدم التفكير في الإصابة هو	-4
………. مقياس ثقتي في الجزء المصاب من الجسم لتلبية احتياجاتي أثناء اللعب هو	-5
……………………………… مقياس ثقتي في مستوى مهارتي/قدرتي هو	-6
Add total and divide by 10 = ………..	
………………………………………… = 10 اجمع الإجمالي واقسمه على	
